# A Rare Case of Pulmonary Embolism in a Patient With Interrupted Inferior Vena Cava and Polysplenia

**DOI:** 10.7759/cureus.22323

**Published:** 2022-02-17

**Authors:** Mahmoud M Mansour, Omar Hussein, Baraa Saad, Alexander Shinn, Tarang Patel

**Affiliations:** 1 Internal Medicine, University of Missouri, Columbia, USA; 2 Pulmonary and Critical Care Medicine, University of Missouri, Columbia, USA

**Keywords:** heterotaxia, polysplenia, ivc, anomalies of inferior vena cava, azygous continuation, interrupted vena cava, sub-massive pulmonary embolism, pulmonary embolism

## Abstract

Interrupted inferior vena cava (IVC) with azygos continuation is one of the anomalies of the inferior vena cava (AIVCs) where venous drainage of the lower extremities is accomplished through a dilated azygos system and is usually accompanied by other congenital malformations such as polysplenia. AIVCs are more common in patients younger than 40 presenting with deep venous thrombosis (DVT). However, pulmonary embolism (PE) in association with AIVCs remains underreported. In this article, we describe a rare case of a 23-year-old male who presented with syncope secondary to sub-massive pulmonary embolism in the setting of an interrupted vena cava draining directly into the azygous vein.

## Introduction

Anomalies of the inferior vena cava (AIVCs) are rare and likely underdiagnosed congenital malformations with an estimated prevalence of 0.6-3% in healthy individuals [1]. AIVCs are frequently associated with other congenital anomalies such as left isomerism and polysplenia. Azygos continuation with interrupted inferior vena cava (IVC) is a common form of AIVCs characterized by the draining of the IVC directly into the azygous vein along with the absence of the hepatic segment of the IVC [2].

Although most AIVCs remain asymptomatic, they are becoming more recognized as a risk factor for deep venous thrombosis (DVT), particularly in the young population [3-5]. However, pulmonary embolism (PE) associated with AIVCs is much less reported in the literature. Herein, we describe sub-massive pulmonary embolism in a young male with an azygous continuation of the IVC with an associated heterotaxy polysplenia syndrome.

## Case presentation

A 23-year-old previously healthy man presented to the hospital with two episodes of passing out while walking in a park. Witnesses reported no jerking movements, tongue biting, or urinary incontinence, and the patient regained consciousness in about 10 seconds. The patient denied previous similar episodes or preceding symptoms such as palpitations, vision changes, or lightheadedness. The only recent change in his health was that, over the past week, he started to feel short of breath with exercise and complained of chest pain when he took deep breaths. He denied any history of tobacco, alcohol, or illicit drug use. He also denied a family history of congenital heart disease or sudden cardiac death.

A physical exam revealed a respiratory rate of 32 breaths per minute, a heart rate of 130 beats/minute, and oxygen saturation of 88% on room air. The lab workup was remarkable for elevated troponin T at 0.12 ng/mL (normal < 0.04 ng/mL) and NT-ProBNP at 1400 pg/mL (normal < 125 pg/mL). The complete blood count and comprehensive metabolic panel were both within normal values. An electrocardiogram showed sinus tachycardia with no ST or T wave changes. A chest X-ray did not show any acute findings.

**Figure 1:**
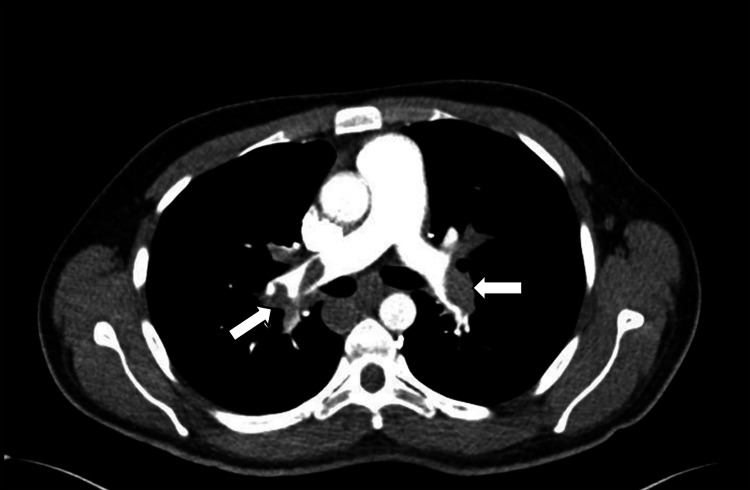
Axial view of CTPA in the arterial phase showing filling defects (acute PE) involving the left and right main pulmonary arteries (arrows). CTPA: computed tomography pulmonary angiogram, PE: pulmonary embolism.

**Figure 2:**
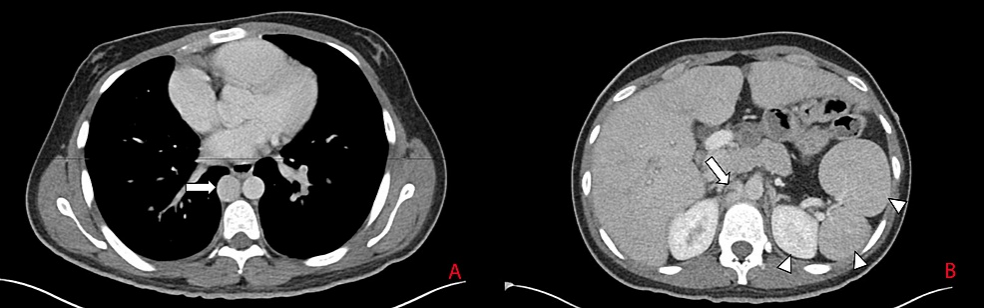
(A) Axial view of CTPA in the venous phase showing the enlarged azygos vein (arrow) coursing alongside the descending aorta. (B) Axial view of CT scan of the abdomen with IV contrast demonstrating multiple spleens (arrow heads). Also note the anastomosis between the IVC and the azygos vein (arrow). CTPA: computed tomography pulmonary angiogram, CT: computed tomography, IV: intravenous, IVC: inferior vena cava.

**Figure 3:**
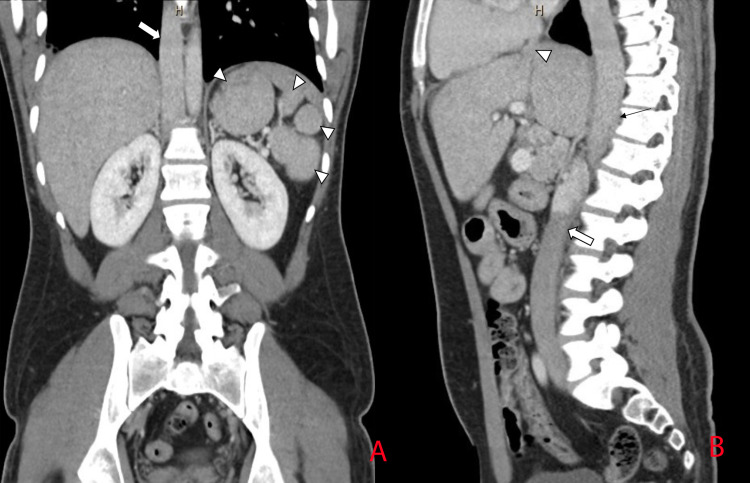
(A) Coronal view of CT scan of the abdomen with IV contrast showing multiple spleens (arrow heads) and the dilated azygous vein (arrow) as a direct continuation of the IVC. (B) Sagittal view of CT scan of the abdomen showing the IVC (wide arrow) draining directly into the dilated azygous vein (thin arrow). Notice the absence of the hepatic segment of the IVC and the hepatic veins draining directly into the right atrium (arrow head). CT: computed tomography, IV: intravenous, IVC: inferior vena cava.

In light of hypoxia, tachycardia, and pleuritic chest pain, PE was a primary concern. A computed tomography (CT) with pulmonary angiogram (CTPA) was obtained, and it revealed bilateral PEs in the main pulmonary arteries (Figure 1). The CTPA scan also showed an incidentally dilated azygous vein concerning an IVC anomaly (Figure 2A). Subsequently, a CT of the abdomen and pelvis with IV contrast was obtained and showed interruption of the IVC, which continued directly into a dilated azygous vein, as well as polysplenia and non-rotation of the small intestine, manifested as a left-sided appendix and right-sided ligament of Treitz (Figures 2B, 3A, 3B). An echocardiogram was obtained and revealed dilation of the right ventricle suggestive of right-sided heart strain. The lower limbs' ultrasound was negative for deep vein thrombosis. A diagnosis of sub-massive (intermediate-high) PE was made.

The patient was started on therapeutic anticoagulation. The interventional radiology team discussed mechanical thrombectomy with the patient; however, the patient opted to forgo the intervention. After four days of hospitalization, the patient made a prompt recovery with improvement in shortness of breath and resolution of hypoxia and tachycardia. The patient had no personal or family history of venous thromboembolism (VTE). He had no history of smoking, immobility, or recent surgery. Follow-up hypercoagulability workup with factor V Leiden, prothrombin mutation, protein C or S deficiency, and lupus anticoagulant was unremarkable. The patient was discharged with a plan for lifelong anticoagulation, given the ongoing risk of VTE.

## Discussion

Development of IVC occurs during the sixth to eighth weeks of embryogenesis. It evolves from three primitive veins: supracardinal, posterior cardinal, and subcardinal. These develop temporarily, then regress, and finally anastomose to form the four segments of the IVC: hepatic, suprarenal, renal, and infrarenal. During this transformation of the IVC, numerous anomalies may occur [6]. Interruption of the IVC with azygos continuation occurs when the subcardinal-hepatic anastomosis fails to form, resulting in congenital absence of the hepatic segment of the IVC. Consequently, the IVC continues superiorly as a dilated azygos vein which eventually empties into the superior vena cava. Azygos continuation of the inferior vena cava anomaly has an estimated 0.6% prevalence [1].

The presence of AIVCs has been associated with other anomalies, such as heterotaxia syndrome. Heterotaxia is when the internal thoracoabdominal organs demonstrate abnormal arrangement across the left-right axis of the body. A common manifestation of heterotaxia is nonrotation of the small intestine, which can manifest on imaging as a left-sided appendix and right-sided ligament of Treitz [7]. Heterotaxia can be further classified into heterotaxia with polysplenia or heterotaxia with asplenia. Interestingly, polysplenia itself has been associated with venous thromboembolism, presumably related to thrombocytosis [8].

In the majority of cases, AIVCs remain asymptomatic due to collateral circulation in the abdomen [9]. However, despite the compensatory collaterals, decreased drainage of the lower extremities leads to increased venous pressures and stasis, thus increasing the risk of thrombosis. Retrospective studies estimated the presence of AIVC in young patients (<30 to 40 years) presenting with DVT to be 5-9.5% [3-5]. However, these estimates may be underrepresented because of incomplete radiological evaluation due to insufficient knowledge of these anomalies and their association with venous thromboembolism [10].

On the other hand, PE in patients with AIVCs is exceedingly infrequent in the literature, and there are only a few reports describing the potential association [11-13]. The lower frequency of PE compared to DVT in patients with AIVCs could be attributed to the clots being ensnared in the collateral veins of the IVC that develop due to the interruption [5]. There are no studies, however, that evaluate the prevalence of AIVCs in young patients presenting with pulmonary embolism, and it remains unclear how much less likely those with AIVCs are to develop PE compared to those with DVT only.

## Conclusions

Although rare, AIVCs should be considered as risk factors in healthy young patients presenting with idiopathic PE. In addition to the thrombophilia workup, it is reasonable to consider a contrast-enhanced CT scan of the abdomen to evaluate for AIVCs. It remains unclear if lifelong treatment with anticoagulation is necessary for this patient population. However, it may be considered given the ongoing risk of venous stasis and thrombosis and the potentially catastrophic outcomes of PE.
